# Improved Polymer Membrane for Textile Zinc-Ion Capacitor

**DOI:** 10.3390/polym17222995

**Published:** 2025-11-11

**Authors:** Sheng Yong, Sasikumar Arumugam, Stephen Paul Beeby

**Affiliations:** Centre for Flexible Electronics and E-Textiles, University of Southampton, Southampton SO17 1BJ, UK; s.arumugam@soton.ac.uk (S.A.); spb@ecs.soton.ac.uk (S.P.B.)

**Keywords:** e-textile, energy storage device, zinc-ion capacitor

## Abstract

This work presents the design, fabrication and characterisation of an improved textile energy storage device implemented in a single layer of polyester cotton and silk fabric. To achieve this, the energy storage device has evolved from an electrical double-layer (EDL) supercapacitor to a zinc-ion supercapacitor (ZHSC) with an optimised co-polymer membrane containing a polyethene oxide (PEO) additive and a polyvinylidene (PVDF)-based organic electrolyte. The flexible textile ZHSC achieved an areal capacitance of 159.5 mF cm^−2^ and an energy density of 52.3 µWh cm^−2^ (increasing by a factor of 4 and 1.8, respectively, on the previous work) with a power density of 0.27 mW cm^−2^ and good bending stability.

## 1. Introduction

People are becoming increasingly familiar with wearable electronics, and e-textiles are a growing platform technology for wearables driven by technical improvements, increasing consumer interest and the development of applications across a wider range of industries [1]. Most e-textiles require electrochemical power supplies, and conventional batteries are typically used. However, these are bulky, rigid and incompatible with the flexible and conformable properties of the textile. The textile can serve as an energy storage device [2], but meeting the requirements of high energy and power densities, suitable mechanical flexibility and robustness, cost-effectiveness and sustainability is extremely challenging. Among the many potential energy storage devices, the zinc-ion hybrid supercapacitor (ZHSC) avoids the use of hazardous electrolytes, provides long-term cycling stability in contrast to secondary batteries, improves the energy density and lowers the leakage current compared with the electrical double-layer (EDL) supercapacitor [3]. ZHSCs overcome the capacitance/energy storage limitation of symmetrically configured EDL supercapacitors and enhance the power density that can be delivered from secondary batteries [4]. A ZHSC consists of a porous and conductive carbon cathode, a zinc metal anode and an ion-conducting electrolyte. It stores electrical energy based on faradaic or redox reaction mechanisms and electrostatic adsorption/desorption or EDL mechanisms [5]. A textile ZHSC will provide an embedded power supply for e-textile systems with improved electrical properties, minimising the surface area required and making the device as unobtrusive as possible.

The ZHSC is one of the hybrid metal-ion energy storage devices. Metals such as sodium [6], potassium [7], lithium [8], aluminium [9] or zinc [10] have been demonstrated in textile capacitors/supercapacitors as device anodes. These metal anodes were separated by a membrane with a porous carbon or metal oxide cathode; both electrodes were covered with an ion-conducting aqueous or organic electrolyte that transports metal ions in between to trigger a redox reaction [11]. Among these metals, zinc is a material that is nature abundant [12], low cost [13], usable with a non-flammable electrolyte [14], safe [15] and mechanically flexible [16]. It is a low-toxic material and presents no threat to human life [17]. Zinc has a high theoretical capacity of 5849 mAh cm^−2^ or 819 mAh g^−1^, and a zinc anode also works with a less hazardous and non-flammable aqueous or organic electrolyte solution [18]. In a ZHSC, the faradaic reaction mechanisms at the anode contribute to the energy density of the device, and the electrostatic adsorption/desorption or EDL mechanisms at the cathode contribute a high power density and rapid charge/discharge rates and extend the ZHSC’s cycling lifetime beyond what is possible for typical secondary batteries [19]. A ZHSC with an aqueous electrolyte can be charged to 1.8 V, given zinc’s redox potential, which is higher than that for an EDL supercapacitor with symmetrical activated carbon electrodes with the same aqueous electrolyte (0 to 1 V) [20]. Alternatively, a wider and stable voltage window of 0–3 V can also be achieved in an EDL-type device with a water-in-salt electrolyte containing lithium bis (trifluoromethanesulfonyl)imide [21]. However, the toxicity of the electrolyte material limits the suitability of this in wearable e-textile applications. These advantages make zinc an attractive material for a textile energy storage device design.

Previously, Zhang et al. [22] reported flexible ZHSCs with an activated carbon cloth cathode and an electroplated zinc/carbon cloth anode with a polyacrylamide/ZnSO_4_ aqueous electrolyte. Both cathode and anode were prepared with different pieces of the same carbon cloth. The reported ZHSC achieved an areal capacitance of 2.43 F cm^−2^ and an areal energy density of 1.35 mWh cm^−2^ at 1 mW cm^−2^. Li et al. [23] demonstrated a ZHSC with reduced graphene oxide/carbon nanotube/polypyrrole (GCP-op) cotton fabric as the cathode, zinc metal as the anode and a polyacrylamide/ZnSO4 aqueous electrolyte. The proposed ZHSC demonstrated a specific capacitance of 8.39 F·cm^−2^ and an energy density of 0.158 mWh cm^−2^ at 0.5 mW cm^−2^. In both cases, the ZHSCs were assembled with multiple fabric layers and special carbonised cotton fabrics with complex treatments, hindering the device’s scalability. In textile ZHSC design, it is advantageous to integrate the functional cathode, zinc anode separator and electrolyte into a single textile layer to reduce the device’s complexity and improve its scalability.

A textile ZHSC requires a separator membrane to block zinc dendrites and prevent physical short circuits while facilitating the ion exchange between the cathode and anode [24]. This separator membrane also needs to be mechanically flexible, resist chemical degradation from the electrolyte and redox reaction and have high electrolyte uptake and material porosity [25]. A ZHSC, zinc-ion battery or zinc–air battery generally uses glass microfiber paper [26], cellulose [27] or electrospun polyacrylonitrile (PAN) fibre [28] as separators, with the cathode and anode implemented on different substrates. In a textile device design, these porous layers have drawbacks as they can only physically bond with textile fibres (glass fibre and PAN fibre) [29] or are sensitive to water molecules contained in moisture that causes structural film damage and results in device short circuits (cellulose substrate) [30]. Another approach is to form a porous membrane in the structure of the textile to hold the electrolyte and separate the cathode and anode. Previously, Yong et al. [31] reported a supercapacitor fabricated within a single polyester cotton textile layer with an ethylene vinyl acetate–poly methyl methacrylate (EVA-PMMA) porous polymer membrane formed in the textile. Here, the PMMA provides the separator membrane with a porous structure, and the EVA enhances its mechanical durability and adhesion to textile fibres. The flexible EDL capacitor achieved a capacitance of 38.2 mF cm^−2^. However, the EVA-PMMA porous polymer has an issue with its affinity with a zinc-ion electrolyte and fails to prevent zinc dendrite formation. These can be reduced by combining a polyethene oxide (PEO) polymer in the polymer networks of the separator membrane [32] and introducing polymers such as PVDF in the electrolyte [33]. PEO is a mechanically flexible, synthetic, non-ionic, hydrophilic and biocompatible polymer that improves the separator membrane’s affinity with the electrolyte and prevents side reactions and solvent evaporation in ZHSCs and zinc-ion batteries [34].

This work presents the fabrication and characterisation of a novel textile ZHSC using a solution-processed carbon cathode and zinc anode, with a co-polymer membrane and organic polymer electrolyte, all integrated into a single layer of polyester cotton textile. The fabrication process is scalable and compatible with processes used in the textile industry. In comparison, the areal capacitance of the reported textile ZHSC is 290% higher than an equivalent textile EDL supercapacitor reported [31], indicating a scalable route for realising high-performance energy storage devices in textiles.

## 2. Materials and Methods

### 2.1. Material

Activated carbon YP-80F from Kuraray Chemical (Tokyo, Japan), EVA binder beads (vinyl acetate 12 wt. %), PMMA powder (with an average Mw of 120,000 by GPC), PEO powder (with an average Mw of 8000 by GPC), carbon black conductive additive (<100 nm particle size), zinc powder, zinc trifluoromethanesulfonate, 1,2,4-trichlorobenzene, ethanol, cyclohexanol and isoamyl acetate were acquired from Sigma-Aldrich (Gillingham, UK). The polyester cotton (denoted as PC) had fibre diameters of 12 µm (polyester) and 15 µm (cotton), 16.5 ends per inch (9.05 picks per inch) and a weight of 27.1 g cm^−2^ and thickness of 250 µm.

### 2.2. Fabrication of Polyester Cotton Textile with Improved Polymer Membrane

The fabrication process of the membrane textile (denoted as PC 217) involved screen printing, phase inversion and a curing process and has been reported in previous articles [31]. In comparison with the reported membrane composition, the membrane polymer solution contains hydrophilic polymer PEO with a different solvent, cyclohexanol. The combined polymer blends include EVA beads (vinyl acetate 12 wt.%), PEO powder and PMMA powder at a weight ratio of 2/1/7. 3 g of the polymer blend dissolved in a solvent mixture of 2.5 mL of cyclohexanol and 2.5 mL of isoamyl acetate. The membrane polymer solution was heated on a hot plate at 80 °C with a magnetic stirring bar rotated at 300 rpm for 4 h until the solution became clear. The membrane polymer solution was then screen printed on top of the polyester cotton textile with a screen mesh thickness of 40 µm, printing gap of 0.8 mm, printing pressure of 5.5 kg and speed of 10 mm s^−1^. After printing, the membrane polymer-coated cotton textiles were treated with a phase inversion process by immersing the samples in ethanol within a sonication bath for 15 min. The samples were then cured under vacuum for 2 h at room temperature. After the curing process, the remaining co-polymer forms a membrane layer of about 80 µm on top of the textile. A similar membrane textile has been reported previously [31]. It was prepared (denoted as PC 37) to enable a comparison with PC 217. The polymer membrane in PC 37 comprised EVA beads with PMMA powder at a weight ratio of 3:7 without the PEO additive.

### 2.3. Fabrication Process of Cathode and Anode

**Figure 1:**
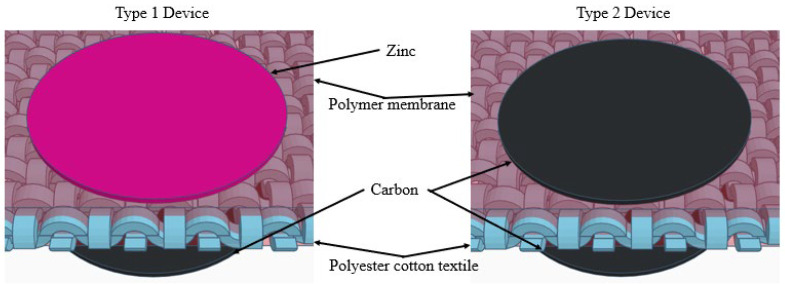
Schematic of type 1 (ZHSC) and type 2 (CC) devices.

The carbon electrode solution contains activated carbon YP-80F, carbon black conductive additive and EVA binder at a weight ratio of 76.5:8.5:15, dissolved in a 1,2,4-trichlorobenzene solvent [10]. The zinc anode solution contained 4.5 wt.% of binder EVA and 95.5wt.% of zinc powder dissolved in 1,2,4-trichlorobenzene solvent. The carbon and zinc solutions were spray-coated on top of the opposite side of the membrane textile through a metal mask with an open area of 0.785 cm^2^ each. The spray nozzle with a diameter of 0.3 mm was located 15 cm away from the fabric, and the spray coating pressure was 25 psi (1.72 bars). Each side of the textile sample was sprayed for 5 s. The area of each textile device was 0.785 cm^2^. During the spray coating process, material mists were formed and adhered to the unmasked area of the membrane textile uniformly, as reported previously. Two types of devices were prepared, shown in Figure 1: the type 1 device had a zinc anode and a carbon cathode (denoted ZHSC), and the type 2 device had a carbon electrode on both sides of the membrane textile (denoted CC). The weight of the active material (carbon) left on a single side of the ZHSC was 2.4 mg cm^−2^, and the total active material left on both sides of the CC was 4.8 mg cm^−2^.

### 2.4. Textile ZHSC Assembly and Testing

The organic polymer electrolyte (OPE) was made by mixing 1 g of ethylene carbonate (EC), 1 g of propylene carbonate (PC), 0.1 g of polyvinylidene fluoride powder (PVDF) and 0.3 M of zinc trifluoromethanesulfonate ionic zinc salt. The organic polymer electrolyte was centrifuged at 4500 rpm for 5 min, followed by heating on a hotplate for 48 h at 90 °C until the mixture became fully transparent. This process ensured all of the zinc salt and PVDF were dissolved in the electrolyte without any gas bubbles. Similarly, an alternative aqueous electrolyte (AE) was prepared for device performance comparison. In the AE, 0.3 M of zinc trifluoromethanesulfonate ionic salt was dissolved in DI water and stirred on a stirrer plate with a magnetic bar until transparent. During the device wetting process, either OPE or AE was vacuum-impregnated into both ZHSC and CC devices at ~25 bar for 20 min. Completed devices were placed under compression using spring-loaded grade 303 stainless-steel current collectors housed within Swagelok PFA tube fittings. The four combinations of devices prepared in this work are shown in Table 1.

### 2.5. Characterisation and Electrochemical Testing of the Membrane Textile and ZHSC

The porosity of the membrane textile was calculated using Equation (1) and the distilled water immersion method [35]. Membrane textile samples were immersed in distilled water under vacuum at ~25 bar for 20 min; the excess distilled water on the surface was removed with a filter paper, and the content of the absorbed distilled water was calculated. The densities of the PC37 textile and PC217 were 1.13 and 1.4 g cm^−3^.

(1) Porosity=MdρdMdρd+M0ρ0×100%

$M_{0}$ and $\rho_{0}$ are the starting weight and density of the PC textile PC217 before vacuum impregnation. $M_{d}$ and $\rho_{d}$ are the weight and density of the distilled water absorbed into the PC, PC 37 and PC 217 textiles.

The electrolyte uptake of the PC, PC 37 and PC 217 textiles was measured by immersing these textile samples into the proposed electrolyte or water under vacuum at ~25 bar for 20 min. The amounts of electrolyte uptake during the ageing test were determined by Equation (2):

(2) uptake%=MeM0×100%

where $M_{e}$ is the weight of the PC, PC 37 and PC 217 textiles with membranes after the wetting process and after 2, 4, 6, 8, 21, 24, 48 and 72 h when left in open air under fume hood extraction at room temperature at 23 °C.

The pore size distribution of the phase-inverted membrane was measured using ImageJ (1.54p) software and the scanning electron microscope (SEM) photograph of the phase-inverted membrane textile.

A Voyager Pro balance was used to measure the weight of the membrane textile and cathode material. A Phenom ProX with an accelerating voltage of 10 kV and with different magnification settings was used to obtain SEM images and EDX results. The electrochemical performance of the ZHSC was measured with an Autolab pgstat101 (Metrohm Autolab, Utrecht, The Netherlands). Cyclic voltammetry (CV) results were obtained at scan rates of 50 mV s^−1^ and voltages varying between 0.1 and 1.8 V. Galvanostatic cycling (GC) tests were obtained at a cycling current of 1 mA cm^−2^ and between 0.1 and 1.8 V unless stated in the text. Electrochemical impedance spectroscopy (EIS) tests of the PC, PC 37 and PC 217 textiles were performed from 100 kHz to 0.1 Hz with an amplitude of 10 mV, using the same OPE and test equipment with the textile ZHSC.

The capacitance, energy and power values of the ZHSC were calculated based on the result from GC with Equations (3)–(5).

(3) C=I×dVdt−1

(4) $$E=I\times \int_{0}^{t}\left(V_{t}+V_{\left(t+1\right)}\right)$$

(5) P=Ettotal

where C is the capacitance; I is the cycling current in the GC test, and dV/dt is the rate of change of voltage between 1.44 V (80% Vpeak) and 0.36 V (20% Vpeak). E is the energy stored in the ZHSC; P is the average power, and t is the time and total time taken to fully discharge the ZHSC.

The mechanical flexibility of the type A and C devices was examined by a cyclical bending experiment. After their initial CV and GC test, the devices were sealed in a polyphenylene bag and cyclically bent around a mandrel with a diameter of 3.2 mm for 2000 cycles, after which the CV and GC tests were repeated. Type B and D devices were not evaluated by the cyclical bending experiment since most of the AE evaporated during testing.

## 3. Results

### 3.1. SEM Imaging

**Figure 2:**
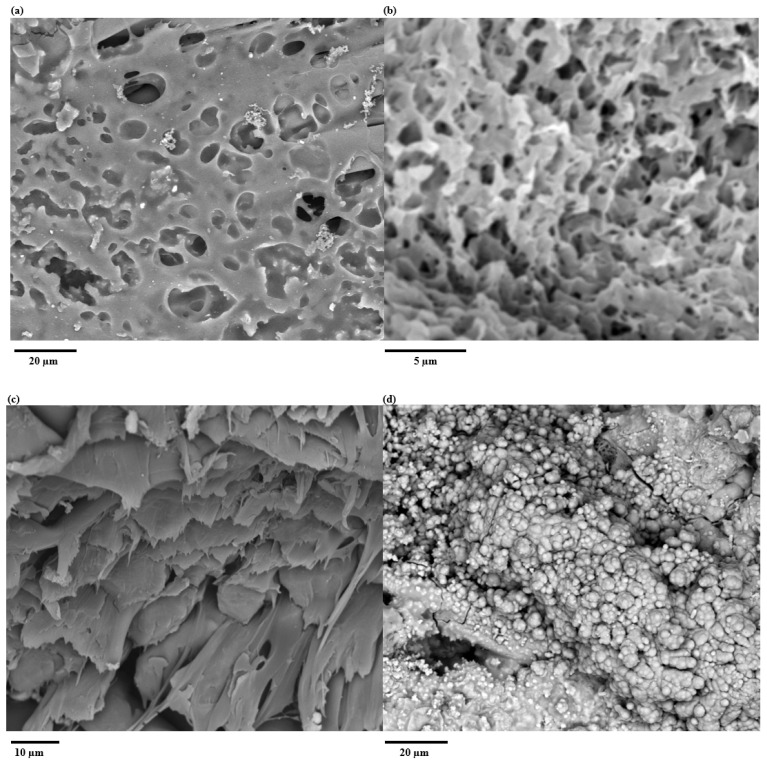

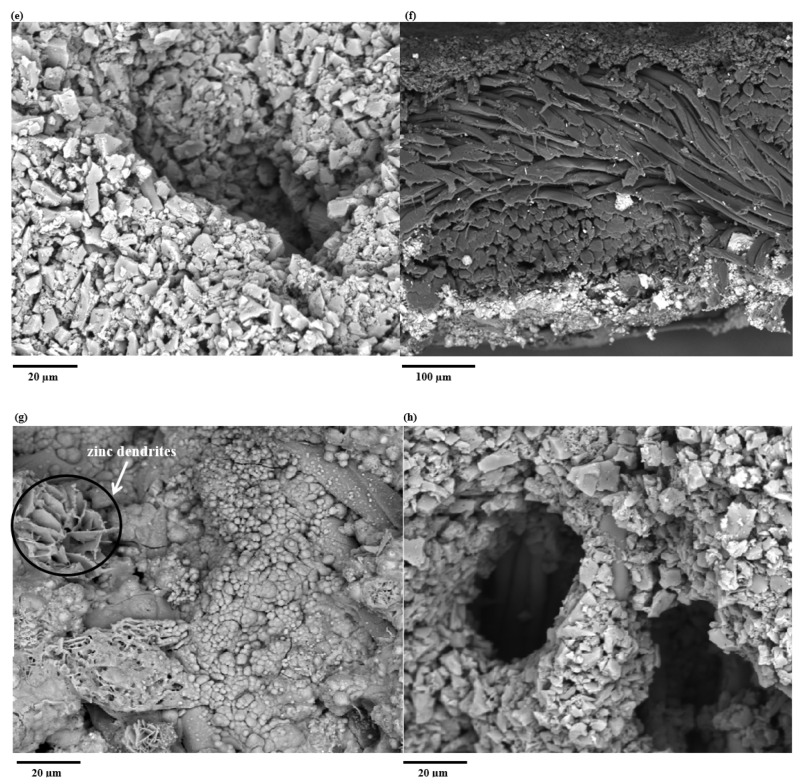
SEM photos of (**a**) plan view of membrane textile (PC 217), (**b**) high-magnification plan view of membrane textile (PC 217), (**c**) cross-section view of membrane textile (PC217), (**d**) plan view of membrane textile with zinc coating before testing, (**e**) membrane textile with a carbon coating before testing, (**f**) cross-section view of textile ZHSC, (**g**) membrane textile with zinc coating after testing showing zinc dendrite, (**h**) membrane textile with a carbon coating after testing showing no change.

The phase-inverted PMMA-EVA-PEO co-polymer separator is shown in Figure 2a,b. The co-polymer composite forms a porous structure made up of polymer lattices with micropores of around 3 µm in diameter, allowing ion movement across the textile ZHSC and CC. It also blocks the physical gap between the textile yarns (Figure 2c) to prevent the anode and cathode from making physical contact with each other causing short circuits. This also creates an even surface on which the electrodes are subsequently spray-coated, enabling uniform carbon and zinc layers on top of the textile. The EVA polymer improves the adhesion between the co-polymer network and the textile yarns and the mechanical flexibility, while the PEO additive co-polymer network enhances the affinity between the OPE/AE and the co-polymer separator. Figure 2d,e show SEM photos of the carbon and zinc coating on top of the PC 217 textile. The SEM photograph in Figure 2f shows the cross-section view through the type A textile ZHSC. The PMMA-EVA-PEO co-polymer membrane located at the centre of the SEM image forms a continuous separator and physically separates the carbon and zinc electrodes on each side of the textile. The total thickness of all textile devices was approximately 350 µm. Figure 2g shows the SEM photograph of the zinc coating on the membrane textile after the cycling test. After 1000 cycles, zinc dendrites grew on top of the zinc particles during the test. These morphological and material changes on top of the zinc particles reduce the reaction area, which is responsible for a reduction in the ZHSC’s energy storage performance. The SEM photograph of the carbon coating on the membrane textile after the cycling test in Figure 2h shows that the OPE did not introduce any morphological or material changes to the carbon particles.

### 3.2. Porosity, Electrolyte Uptake and Ageing Test

**Figure 3:**
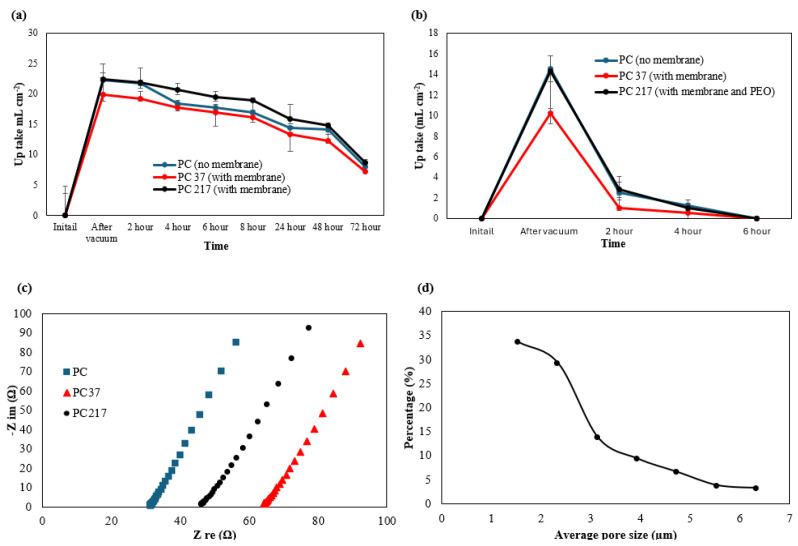
(**a**) OPE uptake and ageing test of PC textile, PC 37 textile and PC 217 textile; (**b**) AE uptake and ageing test of PC textile, PC 37 textile and PC 217 textile; (**c**) Nyquist plots of PC 37 textile and PC textile. (**d**) Average pore size distribution of PC 217 textile.

The PC 217 textile demonstrated the highest porosity of 34.2 ± 5%, which was 9.25% higher than the PC 37 textile (24.9 ± 6%) and 2.43% higher than the PC textile (31.8 ± 4%). It shows the additional PEO additive can increase the electrolyte uptake of the PMMA/EVA polymer membrane in polyester cotton textiles. The electrolyte uptake and ageing test results are shown in Figure 3a. After impregnating the samples with the organic electrolyte used in the electrochemical tests, the PC 217 textile absorbed the highest volume of organic electrolyte solution (22.42 ± 10% ml cm^−2^) followed by the PC textile (22.24 ± 12% ml cm^−2^) and PC 37 (19.89 ± 10% ml cm^−2^); these results are consistent with the measured porosities. These textile samples were stored in air at 23 °C for 72 h to evaluate the electrolyte retention. After 72 h, PC 217 textile maintained 8.67 ± 12% ml cm^−2^ of the OPE, which was greater than the values for both the PC 37 (7.23 ± 13% ml cm^−2^) and the PC textiles (7.95 ± 13% ml cm^−2^). These results indicate the PEO additive increases the uptake of OPE in PC textiles and PMMA/EVA polymer networks (PC 217) and improves its ageing performance against evaporation. To make a comparison between OPE and AE, a similar uptake and ageing test was performed with AE, as summarised in Figure 3b. All of the AE evaporated after 6 h. The combination of the OPE with the PMMA-EVA-PEO co-polymer membrane reduces the electrolyte evaporation rate and hence increases the lifetime of the ZHSC. It also shows that the PC 217 samples can uptake and retain a higher volume of OPE than AE. To investigate the membrane’s performance when used in energy storage devices, the Nyquist plot was extracted from the EIS test of the PC, PC 37 and PC 217 textiles and is shown in Figure 3c. The equivalent series resistance of the PC 217 textile (45.9 ± 16.7% Ω or 0.87 ± 13.2% mS cm^−1^) is lower than that of the PC 37 textile (64.3 ± 18.3% Ω or 0.62 ± 14.2% mS cm^−1^), but higher than that of the PC textile (31.7 ± 15.3% Ω or 1.26 ± 14.6% mS cm^−1^) (characterised close to 100 kHz) at the crossing point of the real axis. This result shows the PEO additive reduces the equivalent series resistance of the PMMA/EVA polymer membrane in the textile due to the increased absorbed electrolyte in PC 217 samples compared with the PC 37 samples.

Figure 3d shows the pore size distribution of the phase-inverted PMMA–EVA–PEO membrane (PC 217 samples). More than 92% of the pores introduced by the membrane have an average diameter less than 5.54 µm, which is less than the median diameter (D50) of the activated carbon powder Yp-80F (5.6 µm [36]) and the mean particle size of zinc powder (7.5 µm [37]). These results show that the co-polymer membrane can block the majority of the material coated on top of the PC 217 samples and effectively prevents them from electrically short circuiting.

### 3.3. Electrochemical Results

**Figure 4:**
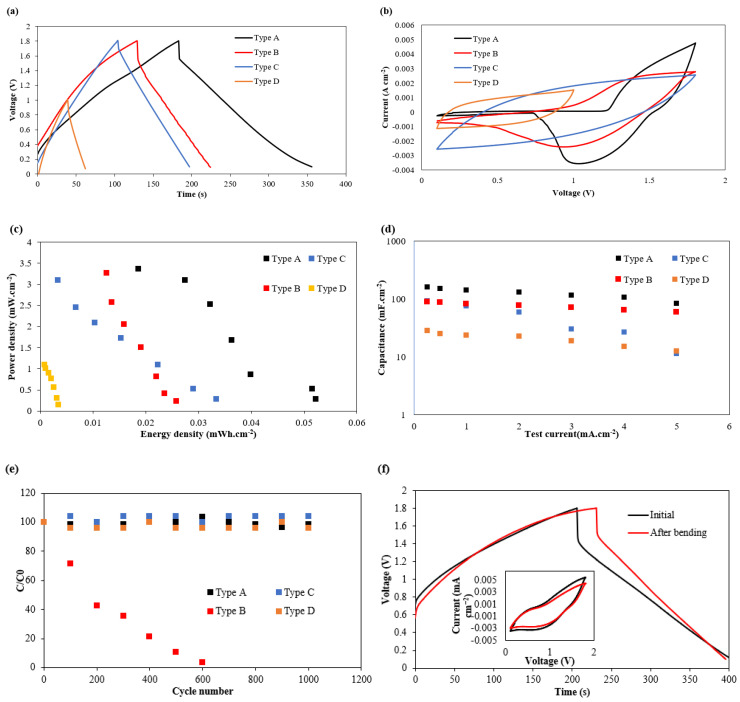
Electrochemical test results of device type A (black), type B (red), type C (blue) and type D (orange). (**a**) GC results with constant cycling current at 1 mA cm^−2^. (**b**) CV test results at a scan rate of 50 mV s^−1^. (**c**) Ragone plot from 0.25, 0.5, 1, 2, 3, 4, 5 mA cm^−2^. (**d**) Capacitance at different current densities. (**e**) Cycling performance over 1000 cycles from the GC test with test current at 2 mA cm^−2^. (**f**) Bending experiment results of the type A device from CV (at 50 mV s^−1^) and GC (at 1 mA cm^−2^) before and after bending (2000 cycles).

Figure 4 shows the electrochemical test results from the A, B, C and D devices, with results being an average from five devices in each case.

Figure 4a shows the GC results between 0.1 V and 1.8 V for types A, B and C and between 0.1 V and 1.0 V for type D supercapacitors. The type A device had the highest areal capacitance (CA) of 110.8 mF cm^−2^, followed by the type B ZHSC (82.4 mF cm^−2^) with the same electrode configuration but a different electrolyte. The areal capacitance of the type A device was higher than that of the symmetrical CC supercapacitor device (50.1 mF cm^−2^) with the same electrolyte. A type D symmetrical CC supercapacitor with the aqueous electrolyte achieved the lowest areal capacitance and operating voltage due to the lower electric potential window of water. These indicate that the use of an asymmetrical electrode configuration (zinc/carbon) in the ZHSC improves the areal capacitance by triggering a faradic reaction that occurs at the zinc metal electrode to store the electrical energy. The use of an OPE also increases the areal capacitance because the membrane textile PC 217 can uptake and retain a higher volume of OPE than AE, and the area of electrode material in contact with the electrode in the type A device is greater than that in the case of the type B ZHSC.

Figure 4b shows the CV test results for type A, B and C supercapacitors between 0.1 V and 1.8 V and type D devices between 0.1 V and 1.0 V. The CV test results for the type A and B devices with zinc/carbon asymmetry electrode set show a redox peak around 0.75 V (type A) and 1 V (type C) when discharging the ZHSC from 1.8 V to 0.1 V, which shows the presence of the zinc anode and confirms the redox reaction in type A and B supercapacitors. The CV test results of type C and D supercapacitors did not show a redox peak; the energy stored in the CC supercapacitors was all based on the electrostatic electrical double-layer mechanism.

The devices’ energy and power density can be extracted from the Ragone plot in Figure 4c, which shows that the type A device achieved the highest energy density at all current densities. When the test current varied from 0.25 mA cm^−2^ to 5 mA cm^−2^, its energy density dropped from 52.3 µWh cm^−2^ to 18.6 µWh cm^−2^ while its average power density increased from 0.27 mW cm^−2^ to 3.36 mW cm^−2^. The type D CC supercapacitor demonstrated the lowest energy and power density at all test currents. This reinforces the previous results, indicating the asymmetrical zinc anode and carbon cathode with the OPE enhances and maximises performance.

Figure 4d shows the areal capacitance variation of the type A, B, C and D devices at different test currents. Type A supercapacitors demonstrated the highest CA of 159.5 mF cm^−2^ when tested at 0.25 mA cm^−2^. This is 156%, 80% and 467% higher than the CA achieved by the type C (62.2 mF cm^−2^), B (88.2 mF cm^−2^) and D (28.1 mF cm^−2^) supercapacitors, respectively. As the test current increases to 5 mA cm^−2^, the CA of the type A device is reduced by 47% (84 mF cm^−2^) and the CA of the type B device is reduced by 87.8% (11.2 mF cm^−2^). This shows that at high cycling currents, the asymmetric electrode design did reduce the capacitance loss due to high test currents.

To study the cyclic stability of the proposed textile supercapacitors, all four types of devices were cycled in an ambient environment (Figure 4e), and their energy storage stability is presented as a ratio of their capacitance during cycling and their initial capacitance at the first test cycle (C0). The two CC EDLC textile supercapacitors (type C and D devices) experienced a capacitance variation of less than 5% over 1000 cycles. The type A ZHSC experienced a capacitance fluctuation of 7.01% over 1000 cycles (approximately 30 h). In the type B ZHSC, more than 90% of its capacitance was lost in the first 600 cycles. This was because of the formation of zinc dendrites that occurs at the anode with an aqueous electrolyte. The use of a combined PE/EC organic electrolyte with a polymer additive successfully reduces this issue, leading to better device cycle stability.

Figure 4f shows the CV and GC plots (inset) of the type A device before (black curve) and after (red curve) cyclically bending the devices around a mandrel with a diameter of 3.2 mm 2000 times. After the bending experiment, the shape of the CV and GC of the red and black curves did not significantly change, with just a small capacitance drop of 5% after the 2000 bending cycles (estimated from the CG plot). This shows that the device’s electrochemical performance is not significantly influenced by the repeated bending and that the binder between the electrode material and the membrane textile can withstand cyclical mechanical strain.

Table 2 shows that the textile ZHSC presented in this work demonstrated higher areal capacitance and energy density than the previous EDLC version, indicating that the textile ZHSC can store more electrical energy than the textile EDLC. The comparison with other devices in Table 2 is less favourable. However, the other devices listed use one or more textile layers in combination with other materials, were assembled and tested in a rigid coin cell configuration and used more-harmful electrolytes. Some of these devices use a textile cathode with a solid zinc metal anode that greatly enhances performance. These other structures, packaging and electrolytes are not compatible with flexile wearable e-textile implementations and are therefore not a direct alternative.

## 4. Conclusions

This work reported for the first time the electrochemical performance and capability of a textile ZHSC fabricated in a single piece of polyester cotton, which, when combined with the polymer-based organic electrolyte, demonstrated significantly improved performance compared with previously reported single-textile-layer devices. The ZHSC were electrochemically stable between 0.1 to 1.8 V and demonstrated an areal capacitance of 159.5 mF cm^−2^, an energy density of 52.3 µWh cm^−2^ and a power density of 0.27 mW cm^−2^. The textile ZHSCs achieved better electrochemical performances with polymer-based organic electrolytes than identical devices with an aqueous electrolyte. The electrochemical performance was also better than that obtained with the symmetrical carbon-only EDLC supercapacitor with the same polymer-based organic electrolytes. ZHSCs with polymer-based organic electrolytes also show good mechanical robustness, being largely unaffected by repeated angular bending as evidenced by a negligible difference in GC and CV results between the bent and non-bent devices. The inclusion of the EVA binder and the spray coating process results in carbon and zinc particles being deposited uniformly and consistently well bonded over the textile substrate.

This work shows the potential for hybrid zinc-ion supercapacitors fabricated in a single layer of polyester cotton textiles to be developed towards a practical solution for powering e-textiles and wearable electronics. Future work will focus on optimising the electrode formulation and fabrication methods of the carbon cathode that lead to higher energy density, improving the porosity and electrolyte affinity of the co-polymer separator membrane in the textiles with increased electrolyte uptake quantity to improve the overall electrochemical performance. In addition, a compatible current collector and encapsulation design will be introduced into the ZHSC design to achieve flexible integrated fabric-based energy storage for powering e-textile devices.

## Figures and Tables

**Table 1:** Device configurations.

Device Type	Textile Electrode Type	Electrolyte Type
A	ZHSC	OPE
B	ZHSC	AE
C	CC	OPE
D	CC	AE

**Table 2:** Supercapacitor performance comparison table.

Ref	Cathode/Anode	Electrolyte	Capacitance (mF cm^−2^)	Energy Density (µWh cm^−2^)	Power Density (mW cm^−2^)
This work	Activated carbon/zinc coating on PC 217	OPE	159.2	52.3	0.27
[38]	Carbonised cotton/zinc metal	2 M Aqueous zinc sulfate	588.8	206.4	0.126
[39]	Activated carbon on carbon cloth/zinc metal	12 M Aqueous zinc sulfate	525.4	241.9	1.76
[22]	Activated carbon on carbon cloth/zinc metal	Aqueous zinc sulfate	2437	1354	1
[31]	Activated carbon coating on PC 217	1M TEABF4 in DMSO	38.2	27.9	0.32
[40]	Cobalt–zinc metal-organic framework textile	Aqueous sulfuric acid	354	196	54.4
[41]	Graphene molybdenum disulfide-coated textile	Aqueous sulfuric acid	1054	58.4	1.604

## Data Availability

The data presented in this study are available on request from the authors. All data supporting this study are openly available from the University of Southampton repository at https://doi.org/10.5258/SOTON/D3351 (accessed on 10 November 2025).

## References

[1] Du K., Lin R.Z., Yin L., Ho J.S., Wang J., Lim C.T. (2022). Electronic textiles for energy, sensing, and communication. IScience.

[2] Zhai S.L., Karahan H.E., Wei L., Qian Q.H., Harris A.T., Minett A.I., Ramakrishna S., Ng A.K., Chen Y. (2016). Textile energy storage: Structural design concepts, material selection and future perspectives. Energy Storage Mater..

[3] Wang L., Peng M.K., Chen J.R., Tang X.N., Li L.B., Hu T., Yuan K., Chen Y.W. (2022). High Energy and Power Zinc Ion Capacitors: A Dual-Ion Adsorption and Reversible Chemical Adsorption Coupling Mechanism. ACS Nano.

[4] Lukatskaya M.R., Dunn B., Gogotsi Y. (2016). Multidimensional materials and device architectures for future hybrid energy storage. Nat. Commun..

[5] Tie D., Huang S.F., Wang J., Ma J.M., Zhang J.J., Zhao Y.F. (2019). Hybrid energy storage devices: Advanced electrode materials and matching principles. Energy Storage Mater..

[6] Zhang H.W., Hu M.X., Huang Z.H., Kang F.Y., Lv R.T. (2020). Sodium-ion capacitors with superior energy-power performance by using carbon-based materials in both electrodes. Prog. Nat. Sci.-Mater..

[7] Wu N.Z., Yao W.J., Song X.H., Zhang G., Chen B.J., Yang J.H., Tang Y.B. (2019). A Calcium-Ion Hybrid Energy Storage Device with High Capacity and Long Cycling Life under Room Temperature. Adv. Energy Mater..

[8] Wang F.X., Liu Z.C., Yuan X.H., Mo J., Li C.Y., Fu L.J., Zhu Y.S., Wu X.W., Wu Y.P. (2017). A quasi-solid-state Li-ion capacitor with high energy density based on LiVO/carbon nanofibers and electrochemically-exfoliated graphene sheets. J. Mater. Chem. A.

[9] Kim Y.I., Kim B., Baek J., Kim J.H., Yoo J. (2022). Hybrid Aluminum-Ion Capacitor with High Energy Density and Long-Term Durability. J. Electrochem. Soc..

[10] Yong S., Wei W.L., Beeby S.P. Full Screen-Printed Zinc-Ion Supercapacitor on Textile for Wearable Electronics. Proceedings of the 2023 22nd International Conference on Micro and Nanotechnology for Power Generation and Energy Conversion Applications (PowerMEMS).

[11] Chojnacka A., Beguin F. (2022). Recent progress in the realization of metal-ion capacitors with alloying anodic hosts: A mini review. Electrochem. Commun..

[12] Vanwinckel H., Mathis J.S., Waelkens C. (1992). Evidence from Zinc Abundances for Dust Fractionation in Chemically Peculiar Stars. Nature.

[13] Dumur F., Beouch L., Tehfe M.A., Contal E., Lepeltier M., Wantz G., Graff B., Goubard F., Mayer C.R., Lalevée J. (2014). Low-cost zinc complexes for white organic light-emitting devices. Thin Solid Films.

[14] Hao J.N., Yuan L.B., Zhu Y.L., Bai X.W., Ye C., Jiao Y., Qiao S.Z. (2023). Low-cost and Non-flammable Eutectic Electrolytes for Advanced Zn-I Batteries. Angew. Chem. Int. Edit..

[15] Xu Z.X., Ma R.J., Wang X.L. (2022). Ultrafast, long-life, high-loading, and wide-temperature zinc ion supercapacitors. Energy Storage Mater..

[16] Long J.W., Han T.L., Lin X.R., Zhu Y.J., Ding Y.Y., Liu J.Y., Zhang H.G. (2023). An integrated flexible self-healing Zn-ion battery using dendrite-suppressible hydrogel electrolyte and free-standing electrodes for wearable electronics. Nano Res..

[17] Hussain S., Khan M., Sheikh T.M.M., Mumtaz M.Z., Chohan T.A., Shamim S., Liu Y.H. (2023). Zinc essentiality, toxicity, and its bacterial bioremediation: A comprehensive insight. Front. Microbiol..

[18] Patil S.J., Chodankar N.R., Hwang S.K., Raju G.S.R., Ranjith K.S., Huh Y.S., Han Y.K. (2022). Ultra-stable flexible Zn-ion capacitor with pseudocapacitive 2D layered niobium oxyphosphides. Energy Storage Mater..

[19] Chen X.D., Zhang H., Gao Y., Liu J.H., Cao X.H., Zhan C.C., Wang S.T., Wang J.Z., Dou S.X., Cao D.P. (2022). Zinc-ion hybrid supercapacitors: Design strategies, challenges, and perspectives. Carbon Neutralizat..

[20] Yang J., Bissett M.A., Dryfe R.A.W. (2021). Investigation of Voltage Range and Self-Discharge in Aqueous Zinc-Ion Hybrid Supercapacitors. ChemSusChem.

[21] Shi C.M., Wang Y., Kulaots I., Zhu H.L., Sheldon B.W. (2024). Water-in-Salt Battery Electrolyte for High-Voltage Supercapacitors: A Fundamental Study on Biomass and Carbon Fiber Electrodes. J. Electrochem. Soc..

[22] Zhang Y.F., Wang P., Dong X.Y., Jiang H.M., Cui M., Meng C.G. (2023). Flexible quasi-solid-state zinc-ion hybrid supercapacitor based on carbon cloths displays ultrahigh areal capacitance. Fund Res..

[23] Li C.W., Hao H.L., Liang J.Y., Zhao B.W., Guo Z.F., Liu G.Z., Li W.Y. (2024). High energy density flexible Zn-ion hybrid supercapacitors with conductive cotton fabric constructed by rGO/CNT/PPy nanocomposite. Nanotechnology.

[24] Liu J.H., Khanam Z., Ahmed S., Wang T., Wang H.T., Song S.H. (2021). Flexible Antifreeze Zn-Ion Hybrid Supercapacitor Based on Gel Electrolyte with Graphene Electrodes. ACS Appl. Mater. Inter..

[25] Zhang Y., Li X., Fan L.S., Shuai Y., Zhang N.Q. (2022). Ultrathin and super-tough membrane for anti-dendrite separator in aqueous zinc-ion batteries. Cell Rep. Phys. Sci..

[26] Maeboonruan N., Lohitkarn J., Poochai C., Tuantranont A., Limthongkul P., Sriprachuabwong C. (2024). Dendrite-free anodes enabled by MOF-808 and ZIF-8 modified glass microfiber separator for ultralong-life zinc-ion hybrid capacitors. J. Energy Storage.

[27] Dmitrenko M., Kuzminova A., Zolotarev A., Selyutin A., Ermakov S., Penkova A. (2023). Nanofiltration Mixed Matrix Membranes from Cellulose Modified with Zn-Based Metal-Organic Frameworks for the Enhanced Water Treatment from Heavy Metal Ions. Polymers.

[28] Qin S.D., Wan C., Xu M.W., Huang J., Chen K., Xu Q.Q., Li S.Z., Zhang F.Z., Guo Y.L., You Y. (2023). An extremely safe and flexible zinc-ion hybrid supercapacitor based on a scalable, thin and high-performance hierarchical structured gel electrolyte. Chem. Eng. J..

[29] Plonka R., Mäder E., Gao S.L., Bellmann C., Dutschk V., Zhandarov S. (2004). Adhesion of epoxy/glass fibre composites influenced by aging effects on sizings. Compos. Part A Appl. Sci. Manuf..

[30] Chen P., Lin X.H., Yang B., Gao Y., Xiao Y., Li L., Zhang H., Li L., Zheng Z., Wang J.Z. (2024). Cellulose Separators for Rechargeable Batteries with High Safety: Advantages, Strategies, and Perspectives. Adv. Funct. Mater..

[31] Yong S., Hillier N., Beeby S.P. (2022). Phase-Inverted Copolymer Membrane for the Enhancement of Textile Supercapacitors. Polymers.

[32] Li H., Ma X.T., Shi J.L., Yao Z.K., Zhu B.K., Zhu L.P. (2011). Preparation and properties of poly(ethylene oxide) gel filled polypropylene separators and their corresponding gel polymer electrolytes for Li-ion batteries. Electrochim. Acta.

[33] Wang F.R., Li L.B., Yang X.Y., You J., Xu Y.P., Wang H., Ma Y., Gao G.X. (2018). Influence of additives in a PVDF-based solid polymer electrolyte on conductivity and Li-ion battery performance. Sustain. Energy Fuels.

[34] Du H., Yi Z.H., Li H.L., Lv W.S., Hu N., Zhang X.Y., Chen W.J., Wei Z.W., Shen F., He H.B. (2024). Separator Design Strategies to Advance Rechargeable Aqueous Zinc Ion Batteries. Chem. Eur. J..

[35] Xu D.M., Teng G.H., Heng Y.Q., Chen Z.Z., Hu D.Y. (2020). Eco-friendly and thermally stable cellulose film prepared by phase inversion as supercapacitor separator. Mater. Chem. Phys..

[36] Kuraray Kuraray Activated Carbon Powder YP-80F, High Power Grade. https://www.cam-energy.com/shop/ku-yp-80-kuraray-activated-carbon-powder-yp-80f-high-power-grade-1801.

[37] Merck GF51714772 Zinc. https://www.sigmaaldrich.com/GB/en/product/aldrich/gf51714772.

[38] Li Z., Huo J., Zhao W., Fan L., Zheng M., Guo S. (2025). Waste cotton fabrics-derived oxygen-rich porous carbon cathode for high-performance zinc-ion capacitors. Mater. Today Commun..

[39] Yao L.L., Koripally N., Shin C., Mu A., Chen Z., Wang K.P., Ng T.N. (2025). Engineering electro-crystallization orientation and surface activation in wide-temperature zinc ion supercapacitors. Nat. Commun..

[40] Islam M.R., Afroj S., Tan S., Eichhorn S.J., Novoselov K.S., Karim N. (2025). Inkjet-Printed Metal-Organic Frameworks for Smart E-Textile Supercapacitors. EcoMat.

[41] Islam M.R., Afroj S., Karim N. (2023). Scalable Production of 2D Material Heterostructure Textiles for High-Performance Wearable Supercapacitors. ACS Nano.

